# Single-Cell Transcriptome Profiling of Immune Cell Repertoire of the Atlantic Cod Which Naturally Lacks the Major Histocompatibility Class II System

**DOI:** 10.3389/fimmu.2020.559555

**Published:** 2020-10-09

**Authors:** Naomi Croft Guslund, Monica Hongrø Solbakken, Marine S. O. Brieuc, Sissel Jentoft, Kjetill S. Jakobsen, Shuo-Wang Qiao

**Affiliations:** ^1^Centre for Ecological and Evolutionary Synthesis, Department of Biosciences, University of Oslo, Oslo, Norway; ^2^Department of Immunology, Institute of Clinical Medicine, University of Oslo, Oslo, Norway

**Keywords:** Atlantic cod (*Gadus morhua*), single-cell sequencing, *GATA-3*, immune system, gene marker

## Abstract

The Atlantic cod’s unusual immune system, entirely lacking the Major Histocompatibility class II pathway, has prompted intriguing questions about what mechanisms are used to combat bacterial infections and how immunological memory is generated. By single-cell RNA sequencing we here report an in-depth characterisation of cell types found in immune tissues, the spleen and peripheral blood leukocytes of Atlantic cod. Unbiased transcriptional clustering revealed eleven distinct immune cell signatures. Resolution at the single cell level enabled characterisation of the major cell subsets including the cytotoxic T cells, B cells, erythrocytes, thrombocytes, neutrophils, and macrophages. Additionally, to our knowledge we are the first to uncover cell subsets in Atlantic cod which may represent dendritic cells, natural killer-like cells, and a population of cytotoxic cells expressing *GATA-3*, a master transcription factor of T helper 2 cells. We further identify putative gene markers for each cluster and describe the relative proportions of each cell type in the spleen and peripheral blood leukocytes. Of the major haematopoietic cell populations, the lymphocytes make up 55 and 68% of the spleen and peripheral blood leukocytes respectively, while the myeloid cells make up 45 and 32%. By single-cell analysis, this study provides the most detailed molecular and cellular characterisation of the immune system of the Atlantic cod so far.

## Introduction

Teleosts, accounting for nearly half of all extant vertebrates (1), demonstrate an extraordinary level of diversity within their habitat, morphology, physiology, behaviour, and in the genetic repertoire of their immune system (2–4). Whole genome sequencing of the Atlantic cod (*Gadus morhua*) and other Gadiform species revealed that genes encoding Major Histocompatibility class II (MHCII) were missing, along with the absence of the entire CD4+ T cell component of the adaptive immunity (5, 6). For the first time, it was demonstrated that this classical immune pathway can no longer be considered the hallmark of vertebrate immunity (4, 6, 7). Why Atlantic cod and its relatives have lost the MHCII pathway is not known; however, several putative past biological scenarios have been suggested (4, 8). Additional peculiarities mark the Atlantic cod immune system, including extreme expansion of MHC class I (MHCI) genes as well as gene losses and expansions within the innate immune system (3, 5, 9, 10). Further, Atlantic cod studies have reported a low to modest response by specific antibodies following pathogen exposure but a consistently high level of natural IgM (11, 12). Since the Atlantic cod and codfishes demonstrate such an interesting immune system, understanding the workings of an immune system that naturally lacks CD4+ T cells is an evolutionary intriguing question, as well as providing insights into the flexibility of the vertebrate immune system. Moreover, a better grasp of the Atlantic cod immune system would also be beneficial for improved management of cod stock and potential cod aquaculture where infectious disease is a challenge (13, 14).

Traditionally, immune cells are characterised using the unique combination of cell markers present on the cell surface, but for non-model organisms a lack of specific antibody-based reagents makes this approach difficult. Advances in next-generation sequencing technologies allow for a closer examination of biological systems without the need for existing antibodies. The use of single-cell RNA sequencing (scRNA-seq) enables examination of the global mRNA content of thousands of individual cells, and thus facilitates a more detailed characterisation without the need of any pre-existing knowledge. Cell types can be clustered computationally using bioinformatic tools according to their transcriptional activity, and by analysing the transcriptional fingerprint in comparison to an annotated genome cell types and functions can be assigned (15).

A combination of microscopy and *in vitro* functional studies has already identified some immune cell types in Atlantic cod, while whole genome and transcriptome sequencing have led to the identification of putative cell markers. The functional assignment and cell markers of cytotoxic CD8+ T cells (CD8, TRGC1, TNFSF11, EOMES, TCR, CD3), B cells (IGLC2, IgM, CD79), natural killer (NK)-like cells (LITR/NITR, B3GAT1), cells with granules and perforin activities (PERF1, UNC13D), monocytes/macrophages (IL34), and neutrophils (MPO) have been described to some extent within the Atlantic cod (5, 16–19). Additional cell types found in the blood and organs of related teleost species might also be expected in the Atlantic cod, including thrombocytes (20), non-specific cytotoxic cells (NCCs) (21–23) and dendritic cells (DCs) (24, 25). However, the relative proportion of these verified and putative immune cell subsets and an overall assessment of the cellular functions are still lacking. Further, cell type characterisation by means of single-cell RNA sequencing will reveal candidate markers for each cell type which in turn could be used in the development of Atlantic cod antibodies.

In this study, we report an in-depth characterisation of cell types found in immune tissues and organs (*i.e.* spleen and blood) of Atlantic cod by using single-cell RNA sequencing. Gene expression profiling of over 8,000 individual peripheral blood leukocytes (PBLs) and spleen cells combined with conventional morphological microscope studies resulted in the characterisation of 13 distinct cell subsets, of which 11 are likely immune cell populations. Additionally, we identify putative gene markers for each of these cell clusters and provide for the first time, as far as we know, a systematic overview of the relative frequencies of these cell populations in the blood and spleen. Six major cell populations, including the T cells, B cells, erythrocytes, thrombocytes, neutrophils, and macrophages, are shown to make up 94 and 98% of haematopoietic cells in the spleen and PBLs respectively. From these six groups, the lymphocytes make up the majority of cells at 55 and 68% of the spleen and PBLs respectively, while the myeloid cells make up 45 and 32%. In addition, we describe less abundant cell populations which may represent dendritic cells and natural killer cells, as well as a population of cytotoxic cells expressing *GATA-3* which we propose to be a type of innate lymphocyte cell. Our study clearly demonstrates the power of using single-cell RNA sequencing for molecular and cellular characterisation of the immune system in non-model organisms and is a valuable resource for development of antibodies towards the specific Atlantic cod immune cell subsets for future functional studies.

## Methods and Materials

### Atlantic Cod Sampling

The Atlantic cod specimens used in this study originate from the NOFIMA national breeding programme of Atlantic cod (Norway, Tromsø). They all come from one single breeding family (bred from one female and two males) and supplied as juveniles to the NIVA Research Facility at Solbergstrand (near Oslo), Norway where they were reared for approximately one year. The water temperature was kept at an average of 8°C (*e.g.* following the seasonality of the water temperature in this region), with salinity at 34 PSU, and the light conditions were 12:12 h light:dark (L:D) throughout the year. The fish were fed with Skretting cod pellets and checked twice a day. Blood and spleen samples were taken from two specimens of non-vaccinated, 2-year-old Atlantic cod; one male (fish 1, 47 cm, 0.99 kg) and one female (fish 2, 52 cm, 1.77 kg). Tissue sampling was conducted after the fish were killed, which took place within seconds of capture by cranial concussion. Neither fish showed visible signs of infection on skin, gills, fins or internally. Blood samples were collected from the *vena caudalis* with heparinised syringes. The spleens were removed and placed in Leibovitz L-15þ [L-15 (BioWhittaker) adjusted to 370 mOsm by adding 5% (v/v) of a solution consisting of 0.41 M NaCl, 0.33 M NaHCO3, and 0.66% (w/v) D-glucose] and transported on ice. Spleen cell suspensions were obtained by gently forcing the tissue through a cell strainer (Falcon, 100 µm). Blood samples of 0.7 ml were diluted in L-15þ to a total volume of 5 ml. The blood cell suspensions were placed on discontinuous Percoll gradients (3 ml 1.070 g/ml overlaid with 2.5 ml 1.050 g/ml) and centrifuged for 40 min at 400×g and 4°C. A peripheral blood leukocyte (PBL) fraction was collected from the interface of the two Percoll densities, including the downward density layer, and washed twice by diluting the suspension in L-15þ and centrifuging at 300×g for 7 min at 4°C. All cells were kept in regular microcentrifuge tubes to minimise any cell loss and kept on ice at all times.

The rearing and sampling are performed according to animal welfare regulations and approved by the Norwegian authorities (FOTS ID 12336).

### Sorting of Cell Populations by Flow Cytometry

Spleen, blood, and PBL suspensions were further separated into sub-populations on a FACS Aria II flow cytometer (Flow Cytometry Core facility at Oslo University Hospital) gated on the forward scatter (FSC, cell size) *vs* side scatter (SSC, granularity) plot. The sorted populations were examined by microscopy after cytospin and staining.

### Staining of Sorted Cell Populations

Immediately after sorting, 10,000 cells in 15 µl PBS buffer were added to a Cytospin carrier and subjected to centrifugation onto glass slides (80 g for 3 min). Slides were then air-dried and stained either next day or stored at −20 C until fixation and staining. The slides were subjected to routine haematoxylin-eosin (HE) staining. In short, slides of cells were briefly stained with haematoxylin and then by eosin solution, followed by alcohol dehydration. Some cells were also stained for peroxidase activity with the iVIEW DAB detection kit (Roche) according to the manufacturer’s instructions.

### Cell Populations Sent for scRNA-Seq

An overview of the sample origin and the cell populations sent for scRNA-seq using the droplet based scRNA-sequencing (Drop-seq) protocol (15) can be seen in **Supplementary Table 1**. From fish 1 and fish 2, we sequenced unsorted spleen and sub-populations of potential interest from flow-sorted spleen (S3, containing a large myeloid cell population) and the blood (B1, containing a large lymphocyte population). In fish 2 we further extended our investigation into additional populations, including PBLs and flow-sorted populations from PBL: P1 (containing mostly lymphocytes), P2 (containing mostly lymphocytes and thrombocytes), and P3 (containing mostly myeloid cells).

### scRNA-Seq With Drop-Seq

The protocol and reagents used closely followed the protocol written by the McCarroll laboratory, which is an amended version of the method used by Macosko et al., 2015. Briefly, the cell suspension was fed through a droplet generator (Dolomite, UK) that encapsulated a single cell and a barcoded bead in a water-in-oil droplet with a diameter of approximately 80 μm. All the beads contain a primer with a common “PCR handle” sequence to enable PCR amplification. Each individual bead contains 10^8^ primers with the same “cell barcode” but also contains unique molecular identifiers (UMIs), thus enabling the transcripts to be digitally counted at a later stage. A 30-bp oligo dT sequence for the capture of mRNAs is incorporated at the end of the primer. When a cell and bead are enclosed in a droplet the cell is lysed and the poly-dT sequences capture the released mRNA, forming single-cell transcriptomes attached to microparticles (STAMPs). The STAMPs are reverse-transcribed to make cDNA, amplified, and barcoded fragments generated by Tn5-mediated tagmentation. During the post-tagmentation PCR, unique sample barcodes were introduced in the adaptor primers so that samples from different cell populations could be multiplexed in the same sequencing library.

### Quantification of Genes

The libraries were sequenced at the Norwegian Sequencing Centre (Oslo University Hospital), on the NextSeq500 platform with a 75 bp kit, high output mode, with paired end reads. 20 bp was sequenced in Read 1 using a custom sequencing primer (GCCTGTCCGCGGAAGCAGTGGTATCAACGCAGAGTAC) and 60 bp in Read 2 with the regular Illumina sequencing primer. We used the Drop-seq Core Computational Protocol using STAR alignment to map the raw sequencing data to the most recent version of the Atlantic cod genome, gadMor3 (RefSeq accession GCF_902167405.1). A gene of interest, *GATA-3*, was present in gadMor2 (26) but missing in gadMor3, so the *GATA-3* gene sequence was manually added to the gadMor3 assembly fasta file. Reads were then grouped by cell barcode and the unique molecular identifiers (UMIs) for each gene counted, resulting in a digital expression matrix showing the number of transcripts per gene per cell. From each sample, reads from the first 600–5,000 STAMPs (depending on sample size) in decreasing number of reads were included into the next steps for filtering (**Supplementary Figure 2**). Further analysis was performed using R version 3.4.4.

### Cell and Gene Selection

We followed the unsupervised clustering analysis tutorial (27) on the R package Seurat 3.0.2. The data matrix from all samples was merged to create one Seurat object. Cells with a gene count of fewer than 150 or a gene count of more than 1,500 and cells with a total number of molecules of more than 4,000 were filtered away in order to remove low-quality cells and possible cell multiplets (**Supplementary Figure 3**). By excluding genes expressed in less than five cells (among the cells having passed the quality control), 15,273 genes across 8,180 cells were used in the study. An overview of the sample origin, average mapping percentages, included cells, mapped transcripts and genes are shown in **Supplementary Table 1**.

### Cell Clustering and Visualisation

After “LogNormalize” and scaling (with a scale factor 10,000), we ran a principal component analysis (PCA) on the expression of the top 2,000 variable genes. We then used the FindCluster function in Seurat in order to cluster cells based on a shared nearest neighbour (SNN) modularity optimisation result on the top 30 principal components (PCs) (**Supplementary Figure 4**). The resolution for clustering was 0.35. The cell clusters were visualised by the non-linear dimensional reduction method uniform manifold approximation and projection (UMAP).

### Differentially Expressed Genes

All differential expression analyses in this study were performed using FindMarkers embedded in the Seurat package, which performs differential expression based on the non-parameteric Wilcoxon rank sum test. Adjusted p-values were calculated based on Bonferroni correction. In order to be counted as a differentially expressed gene, the gene must be expressed by a minimum of 25% of cells in the cluster. The most significantly differentially expressed genes for each cluster are listed in order of significance (**Supplementary Excel Sheet**).

### Additional Data From Three Wild-Caught Cod

In addition, similar analyses were carried out on unsorted blood and spleen samples from three other Atlantic cod in pilot studies. These samples were taken from wild-caught Atlantic cod from Oslo fjord, taken by direct sampling alongside the project “Monitoring cod health in the inner Oslo fjord”. Data from this pilot experiment accounts for an additional 15,758 genes across 3,744 cells (**Supplementary Excel Sheet** and **Supplementary Figure 5**).

## Results

**Figure 1:**
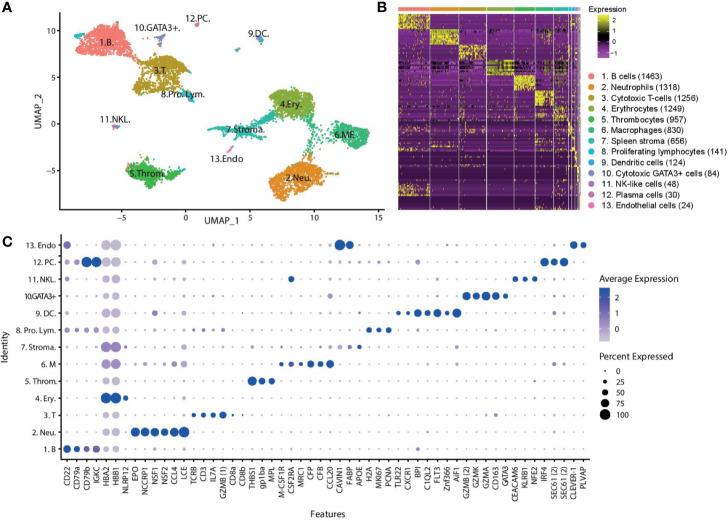
Unsupervised clustering of transcriptomic profiles of Atlantic cod immune cells demonstrates distinct cell populations. **(A)** Visualisation of cell types in two dimensions using UMAP. Putative cell cluster labels are based upon the differential gene expression of known markers in mammals, overall expression pattern and microscopy. **(B)** Heatmap showing differential gene expression, with top 10 differentially expressed genes for each cluster shown (**Supplementary Excel Sheet**). Each row represents a gene, while each column represents a cell. The clusters are shown in descending population size, from B cells to endothelial cells respectively. The size of each cell population is shown in brackets. **(C)** Dot plot showing the putative marker genes across the cell clusters. The size of the dot encodes the percentage of cells within a cluster expressing the gene, while the colour intensity encodes the average expression level of ‘expressing’ cells. *Two *GZMB* and *SEC61* genes are identified (denoted _1_ and _2_ for clarity). **(Key)** 1. B. are B cells, 2. Neu. are neutrophils, 3. T. are T cells, 4. Ery. are erythrocytes, 5. Throm. are thrombocytes, 6. MΦ. are macrophages, 7. Stroma. are spleen stromal cells, 8. Pro. Lym. are proliferating lymphocytes, 9. DC. are dendritic cells, 10. GATA3+. are cytotoxic GATA3+ cells, 11. NKL. are natural-killer like cells, 12. PC. are plasma cells, 13. Endo. are endothelial cells.

Following stringent quality control filtering, we derived a gene expression matrix of 15,273 genes across 8,180 cells (**Supplementary Table 1**). Visualisation of cell types in two dimensions using Uniform Manifold Approximation and Projection (UMAP) (**Figure 1A**) reveals 13 cell clusters with distinct gene expression signatures, with cell cluster sizes ranging from 1,463 cells to the smallest cluster with only 24 cells. Overlapping distribution of cells from the two fish (**Supplementary Figure 6**) supports the robustness of the clusters. Additionally, the clusters formed (**Supplementary Figure 5**), and the top differentially expressed genes expressed by each cluster (**Supplementary Excel Sheet**), are similar to those produced in the pilot study.

To assign a cell identity to each cell cluster we performed differential gene expression analysis. Identified populations include a lymphocyte lineage, with B cells and plasma cells, T cells, cytotoxic GATA3^+^ cells, and putative NK-like cells and a myeloid lineage that includes erythrocytes, thrombocytes, neutrophils, and macrophages (**Figure 1C**). A putative DC population is also described. Additionally, some cells have been classified as spleen stroma and endothelial cells.

The largest cell cluster was identified as the B cell population based on *CD22*, *CD79*, and immunoglobulin genes such as Immunoglobulin Kappa Constant (*IGKC*). A small population of B cells, also expressing immunoglobulin genes and *CD79*, were identified as plasma cells or plasmablasts due to the expression of the transcription factor interferon regulatory factor 4 (*IRF4*) which controls plasma cell differentiation (28). These cells also show high expression of the transport protein *SEC61*, which mediates the transport of proteins across the endoplasmic reticulum. The T cells express the classical T cell genes *TCR*, *CD3*, and Interleukin-7 receptor (*Il7r*). Expression of the cytotoxic protease granzyme B (GZMB_1_) and CD8*α* and CD8*β* confirm this population as cytotoxic CD8^+^ T cells. *CD8α* and *CD8β* are lowly expressed by only a small percentage of cells suggesting that the transcription of these genes may occur in short bursts, or there is a low cell surface expression level. A small group of lymphocytes were differentially clustered from the majority of T and B cells. These cells highly differentially expressed many histone genes, such as histone *H2A*, marker of proliferation Ki-67 (*MKI67*) and proliferating cell nuclear antigen (*PCNA*). This set of genes has greatest expression in actively proliferating cells, and we therefore named these cells proliferating lymphocytes. Some proliferating lymphocytes are more closely aligned to T cells (97 cells), while others are more closely aligned with the B cells (44 cells).

Two additional lymphocyte-related populations were found. Cluster 10, a rare population found in both the PBL and the spleen, is characterized by the expression of *GATA-3* and several cytotoxic enzymes, including granzyme (*GZM*) B_2_, K, and A. *GATA-3* is a transcriptional activator that is shown to play a crucial role in the development of T cells, where it acts as a master regulator of T helper 2 (Th2) differentiation in mammalian species (29, 30), as well as a role in the maturation of innate lymphoid cells (31). We named this population cytotoxic GATA3+ cells.

The cluster labelled as NK-like cells express a somewhat unclear pattern of genes, and it is hard to confidently assign an identify based on classical mammalian markers. These cells differentially express carcinoembryonic antigen-related cell adhesion molecule 6 (*CEACAM6*), killer cell lectin-like receptor subfamily B (*KLRB1*), nuclear factor erythroid 2 (*NFE2*), Colony Stimulating Factor 2 Receptor Alpha Subunit (*CSF2RA*), and the *CD163* molecule. *CEACAM6* is involved in cell adhesion, *KLRB1* is a well-known NK receptor (32, 33), *NFE2* is a transcription factor, *CSF2RA* is a cytokine receptor, and *CD163* is a haemoglobulin scavenger receptor. Collectively, these markers have been affiliated with a range of mammalian cell populations including neutrophils, T cells, NK cells, macrophages, and granulocytes (34, 35). Considering the combined expression profile of these genes, we putatively have named them NK-like cells.

The neutrophils represent the second largest cell population and were identified by the expression of the cytotoxic genes eosinophil peroxidase (*EPO*) and non-specific cytotoxic cell receptor protein 1 (*NCCRP1*), genes involved in phagocytosis, neutrophil cytosolic factors 1 and 2 (*NSF1*, *NSF2*), and chemoattractant genes such as C-C motif chemokine 4 (*CCL4*). The gene classified as *EPO* has a sequence similar to both that of myeloperoxidase, the classic marker of neutrophils, and *EPO*. The most highly expressed gene of the neutrophils is low choriolytic enzyme (*LCE*), a proteolytic enzyme most commonly associated with egg hatching. Zebrafish neutrophils have been shown to express high choriolytic enzyme (*HCE*, also known as *nephrosin*) (36), a related protein with high sequence similarity.

Erythrocytes were identified by the high expression of multiple haemoglobin genes (*HBB*). Erythrocytes also expressed immune related genes, such as NACHT, LRR, and PYD domain-containing protein 12 (*NLRP12*), a potent mitigator of inflammation (37). Expression of thrombospondin-1 (*THBS1*), platelet glycoprotein Ib alpha chain (*GP1BA*), and thrombopoietin receptor (*MPL*) genes was used to identify the thrombocytes.

Macrophages were identified by the marker genes macrophage colony-stimulating factor 1 receptor (M-*CSF1R*), *CSF2RA*, and macrophage mannose receptor 1-like (*MRC1*). This cell population expresses many genes involved in the complement system, such as properdin (*CFP*) and complement factor B (*CFB*), and chemotactic genes, such as C-C motif chemokine 20 (*CCL20*).

Cluster 9 is a small population of cells found mostly in the spleen which express many innate immune genes including toll-like receptor 22 (*TLR22*), the chemokine receptor chemokine XC receptor 1 (*CXCR1*), bactericidal permeability-increasing proteins (*BPI*) and complement genes such as complement C1q-like protein 2 (*C1QL2*). We also observe the expression of the cytokine receptor fms like tyrosine kinase 3 (*FLT3*) and the transcription factor zinc finger 366 (*ZNF366*, also known as *DC*-*SCRIPT*), which have both been linked to differentiation of DCs in mammalian systems as well as in other teleost species (38). Another highly expressed gene in this cluster is allograft inflammatory factor 1 (*AIF1*), a gene which has recently been described in DCs (39). Based on these markers, we have putatively named cells in this cluster DCs.

The cells tentatively named spleen stromal cells express many genes involved in fat metabolism and tissue structure, such as caveolae-associated protein 1 (*CAVIN1*), fatty acid-binding protein (*FABP*), apolipoprotein E (*APOE*). These cells are almost exclusively found in the spleen (94%). The expression profile of these cells is not as clearly differentiated as other clusters, as demonstrated in the heatmap (**Figure 1B**), with a variable expression of genes also found in other clusters. It is possible this cluster of cells is not a ‘true’ cell population and is merely an artefact of ambient RNA captured by beads in empty droplets. A small population of cells branching off from the spleen stroma, population 13, express markers for cell endothelium, including common lymphatic endothelial and vascular endothelial receptor-1 (*CLEVER*-*1*), a protein that is primarily expressed on high endothelial venules and lymphatic vessels (40) where it supports the adhesion and transmigration of lymphocytes (41), and plasmalemma vesicle associated protein (*PLVAP*), an endothelial cell-specific membrane protein (42).

**Figure 2:**
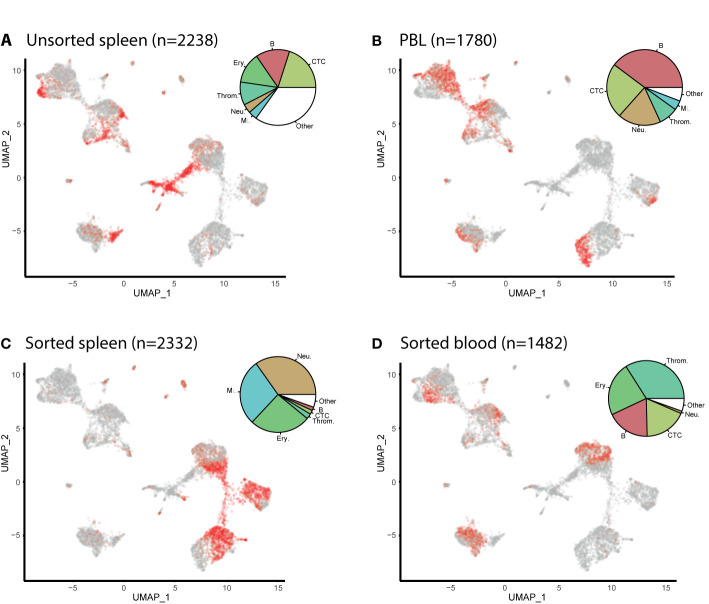
UMAP feature plots show the distribution of cells from Atlantic cod unsorted spleen, peripheral blood leukocytes (PBLs) and flow sorted sub-populations. **(A)** Cells from unsorted spleen. **(B)** Cells from PBL. **(C)** Spleen cells which have been flow-sorted to contain mostly myeloid cells (S3). **(D)** Cells from the blood which have been flow-sorted to contain mostly lymphocytes and thrombocytes (B1). The number of cells per sample is given in brackets (n=). The cells from the named sample are shown in red. The pie charts show the distribution of each cell type in the sample. Resting and proliferating T cells are grouped together, whereas resting and proliferating B cells as well as plasma cells are grouped as B cells. **(Key)** 1. B. are B cells, 2. Neu. are neutrophils, 3. T. are T cells, 4. Ery. are erythrocytes, 5. Throm. are thrombocytes, 6. MΦ. are macrophages, 7. Stroma. are spleen stromal cells, 8. Pro. Lym. are proliferating lymphocytes, 9. DC. are dendritic cells, 10. GATA3+. are cytotoxic GATA3+ cells, 11. NKL. are natural-killer like cells, 12. PC. are plasma cells, 13. Endo. are endothelial cells.

The cells from the unsorted spleen and the PBL samples are found in each cell cluster (**Figure 2**). The largest cell population of the unsorted spleen sample are the spleen stroma cells (27%), classified within the ‘other cells’ in the pie chart, followed by the cytotoxic T cells (22%), the B cells (16%), and then the erythrocytes (13%). In the PBL sample, B cells are the largest cell population (42%), followed by the T cells (25%) and neutrophils (18%). Cells from the sorted spleen S3 sample are populated mostly by neutrophils (35%), macrophages (28%), and erythrocytes (26%). The sorted blood B1 sample contains mostly thrombocytes (34%) and erythrocytes (23%), followed by cytotoxic T cells (19%) and B cells (19%).

The six largest haematopoietic cell populations, including the T cells, B cells, erythrocytes, thrombocytes, neutrophils, and macrophages, make up 94 and 98% in the spleen and PBL respectively. From these major populations, the lymphocytes (including the B cells, plasma cells, T cells, and proliferating lymphocyte populations) make up the majority of immune cells at 55 and 68% of the spleen and PBL respectively, while the myeloid cells make up 45 and 32%.

**Figure 3:**
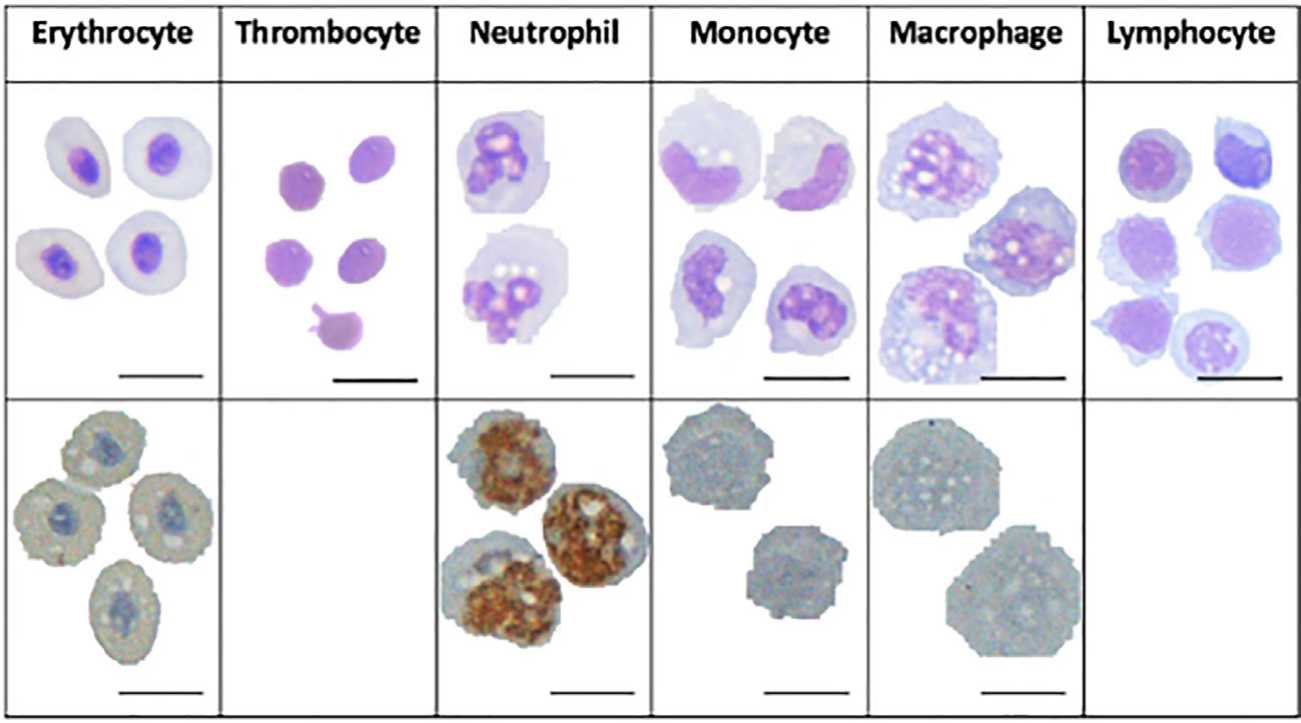
Microscopy images of Atlantic cod immune cells. Cells in the top panel are HE-stained and the cells in the bottom panel are stained with peroxidase. Scale bars 10 μm.

Identification of cells by their transcriptional fingerprint is consistent with the characterisation of the major cell populations by microscopy (**Figure 3**). HE stained S3 sample, which was sorted as a relatively large and granulated population of spleen cells, revealed an apparent maturation of monocytes into macrophages, with a development from a lobed nucleus and few vacuoles to larger nuclei and more vacuoles. Vacuoles could also be seen in the erythrocytes, neutrophils, and in some of the lymphocytes, supporting previous assertations that these are phagocytic cell types. Peroxidase staining of S3 sample revealed peroxidase positive myeloid cells, the neutrophils, and some peroxidase negative myeloid cells, the monocytes/macrophages. The ratio of peroxidase positive and peroxidase negative myeloid cells observed in the S3 population is similar to the ratio of neutrophils and macrophages seen in **Figure 2**.

**Figure 4:**
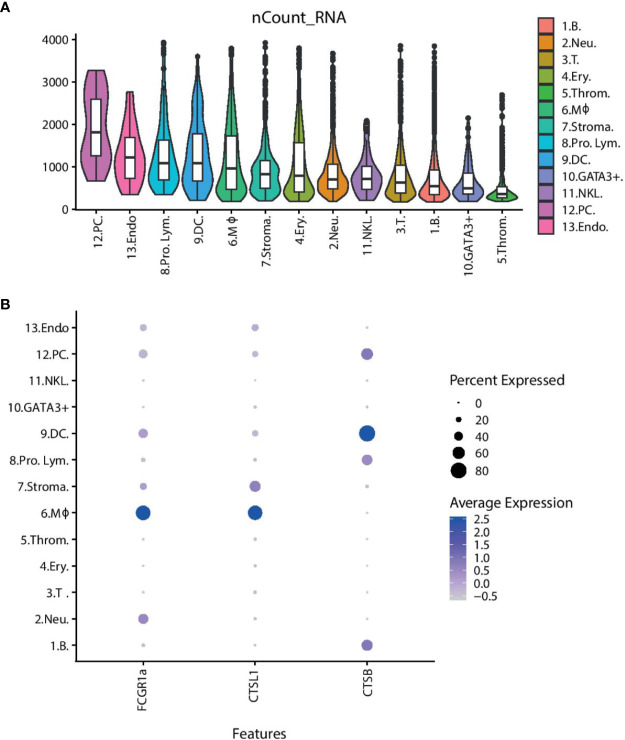
The overall transcriptional activity of each cell type and differential expression of selected genes. **(A)** Violin plots showing the average number of transcripts per cell within the cluster. The overlaid boxplots show the mean and the 25 and 75% percentile of transcripts. **(B)** Dot plot showing the expression of selected genes across the cell clusters. The size of the dot displays the percentage of cells within a class expressing the gene, while the colour intensity encodes the average expression level of ‘expressing’ cells. **(Key)** 1. B. are B cells, 2. Neu. are neutrophils, 3. T. are T cells, 4. Ery. are erythrocytes, 5. Throm. are thrombocytes, 6. MΦ. are macrophages, 7. Stroma. are spleen stromal cells, 8. Pro. Lym. are proliferating lymphocytes, 9. DC. are dendritic cells, 10. GATA3+. are cytotoxic GATA3+ cells, 11. NKL. are natural-killer like cells, 12. PC. are plasma cells, 13. Endo. are endothelial cells.

We next looked at the overall transcriptional activities of the different cell clusters (**Figure 4A**). The average number of transcripts per cell was noticeably high (1,915 transcripts per cell) in the plasma cell group, whereas it was markedly low in the thrombocyte cluster (<500 transcripts per cell). DCs and macrophages also demonstrate a high transcriptional activity (1,200–1,300 transcripts per cell). **Figure 4B** shows the differential expression of cathepsin genes (*CTSB* and *CTSL1*) and an Fc-receptor gene (*FCGR1a*) in these two cell types. MHCI expression is present in all of the cell types as expected; however, poor mapping has resulted in an inconclusive pattern across the cell clusters (**Supplementary Figure 7**).

## Discussion

Despite its lack of MHC-II and CD4+ T cells, the Atlantic cod is able to mount a protective and specific immunity after vaccination (12, 43–46). Interestingly, this protective immunity is poorly correlated with specific antibody responses (46, 47). Indeed, based on these and other molecular observations, it was hypothesised that cod could lack functional MHCII molecules (48) before it was definitely shown by the first assembly of the Atlantic cod genome (5). Despite major genetic losses within the CD4+ pathway and the limited response of specific antibody upon immunisation, in its natural environment cod is not particularly prone to infectious diseases (48). How cod fights bacterial infections and how it acquires immunological memory are puzzling questions that are both interesting biologically and important practically for the cod aquaculture industries. With the near complete lack of antibody-based reagents and immortalised cod immune cell lines these mechanisms have not been fully understood, although some insights have been gained by genome data and transcriptome analyses at the whole organism or organ level (18, 19). To find the exact immune cell composition and the steady-state gene expression of each cell subset is the first step toward a more detailed molecular mapping of the cod immune system.

This study presents an overview of the immune cells found in the Atlantic cod peripheral blood and spleen, using single-cell RNA sequencing and microscopy of both unfractionated and sorted cell populations based on size and granularity. By using unbiased clustering of global transcriptomics, we find 13 different cell populations, with cell identity assigned to these populations based on their unique transcriptional profiles compared with known profiles from mammalian systems. Given the limited number of genes detected in each cell, we restricted our analyses to assign cell population identities, without a more in-depth exploration of the cellular functions of these cell subsets. We identified the most well-known major cell populations such as: the cytotoxic T cells, B cells, erythrocytes, thrombocytes, neutrophils, and macrophages. Overall, these six major cell populations make up 94 and 98% of haematopoietic cells in the spleen and PBL respectively. In the lymphoid lineage, we identified sub-populations of both B and T cells that are actively dividing. In addition, we identified plasma cells as a separate subset in Atlantic cod, supporting previous findings where the presence of plasma cells was suggested by *in situ* hybridisation with immunoglobulin probes (49). To note, a small subset of cells in this cluster were from PBL samples, therefore it is possible that this cluster also contains plasmablasts. Terminally differentiated plasma cells are rarely found in circulation, unlike the more immature plasmablasts (50). This data indicates that Atlantic cod B cells have the capability of end-differentiation into plasma cells despite the lack of CD4+ T helper cells. Future studies are needed to clarify the signalling pathways that are involved in B cell differentiation in cod.

Besides erythrocytes and thrombocytes, based on the top differentially expressed genes, we could clearly delineate the two major phagocytic and cytotoxic subsets within the myeloid lineage; namely macrophages and neutrophils. In mammalian systems, macrophages are important producers of cytokines as well as being antigen-presenting cells, especially in the spleen and lymph nodes (51). However, we could only find a handful of chemokines and no cytokine transcripts in our data. The low sensitivity of detecting transcript for single gene in any given single cell, combined with cytokines being expressed in short bursts only upon activation (52), could explain this particular pattern in our study.

The average number of transcripts that a cell cluster expresses may indicate, with a broad brushstroke, how active the cells are in steady state. Cells which are producing a lot of proteins, proliferating or carrying out multiple tasks, for example phagocytosis and antigen presentation, may be expected to have a higher transcript count than cell populations with fewer “tasks”. Unsurprisingly plasma cells are the most transcriptionally active cell population, with an average transcript count of 1,915 detected transcripts, as this population of cells will be actively producing antibodies. The erythrocytes, neutrophils, NK-like cells, T cells, B cells, and cytotoxic GATA3+ cells have medium levels of transcriptional activity, with approximately 650–1,070 transcripts per cell. These levels represent most likely the steady-state transcriptional activity. The low level of transcriptional activity in thrombocytes is in accordance with the largely absent cytoplasm of these cells. Meanwhile, the DCs and macrophages have a recorded transcript number of roughly 1,200–1,300 per cell. A higher transcript number in these cells compared to the other known phagocytes—the neutrophils, erythrocytes, and B cells—suggest that they may have additional tasks. The macrophages and the DCs are shown to express cathepsin genes (*CTSB* and *CTSL1*) and high affinity immunoglobulin gamma Fc receptor I A (*FCGR1a*). The gene annotation of *FCGR1a* is somewhat misleading since teleost does not have IgG (53). However, as the annotation is based on sequence similarity to annotated genes in other organisms, it is more likely that this gene represents an Ig-Fc binding receptor that could play a role in antibody-mediated uptake of antigens. Cathepsin B and L1 are lysosomal cysteine proteases that play a major role in catabolism of proteins and thus a function in the processing and presentation of antigens *via* MHC (54, 55). The expression of genes involved in antigen presentation coupled with a high transcript number suggests the DCs and the macrophages may act as antigen presenting cells (APCs) in the Atlantic cod. It would be interesting to see if MHCI gene expression is higher in these cell populations and if the expression increases following immune challenge. An initial analysis of MHCI expression in our data is inconclusive, mainly due to the low mapping efficiency. Dedicated efforts using longer reads and tailored bioinformatical tools that can deal with the complexities of MHCI are needed.

Interestingly, the Atlantic cod has evolved MHCI expansion and an unusual repertoire of TLR receptors (3, 5). In addition, novel combination of endosomal sorting motifs was suggested to facilitate a more versatile use of MHCI through cross-presentation and a potential MHCII-like functionality (5, 56). Whether the Atlantic cod is able to mount a cellular immune response that functionally resembles the T helper cells is unknown and remains an intriguing issue for the large group of Gadiform species—all lacking MHCII (6). In our data, the expression of *GATA-3* in a small subset of cells, with an expression profile that indicates close resemblance to the cytotoxic T cells, is an interesting finding. *GATA-3* has been identified and isolated from several species of teleost fish, including zebrafish (57), carp (58), salmonids (59), and in Atlantic cod (60). *GATA-3* expression was detected in surface-IgM-negative lymphocytes in carp (61). Interestingly, the expression of *GATA-3* in Atlantic cod was shown to be increased following stimulation by the T cell-stimulant phorbol 12-myristate 13-acetate (PMA) (60), suggesting the presence of *GATA-3* in activated T cells. In mammals, the transcription factor *GATA-3* plays an essential role in CD4+ T cell development and survival and is necessary for the differentiation of naive CD4+ T cells to T helper (Th) 2 cells (62–64). However, classical T helper cells are absent in Atlantic cod, and this cytotoxic GATA3^+^ cluster lacks the cytotoxic T cell markers that was demonstrated in our data-set (*TCR*, *CD3*, *Il7r*, *GZMB_1_*, *CD8α* and *CD8β*), suggesting that this population may belong to a different lineage than T cells. In mammals *GATA-3* is also central to the development of innate lymphoid cells (ILCs), chiefly the ILC2 lineage (34, 65). Recently, it was shown that *GATA-3* expression was also important for ILC2 in zebrafish (66). ILC2 cells are also known as innate helper 2 cells (67) based on similar cytokine secretion profile. Thus, in summary, the cells described here possibly represent a form of helper ILC. At the same time, these cells also show granzyme expression; indicating possible dual cytotoxic and helper functions. Future studies should look into how this small but intriguing cell subset behaves during immune perturbation, such as immunisation and infection.

In conclusion, using state-of-the-art single-cell sequencing technology on a non-model system, we performed a detailed molecular and cellular characterisation of the Atlantic cod immune system. In addition to describing in more detail the major cell subsets, we also describe for the first time in Atlantic cod, as far as we know, cells that may represent dendritic cells, natural killer-like cells and innate lymphoid cells, as well as suggest that macrophages and dendritic cells may act as antigen presenting cells. Further functional characterisation of these cells is needed to delineate their role in antigen presentation. This work provides an expression profile baseline of the Atlantic cod in a steady state, which lays a foundation for future work with immune system challenge experiments. Future challenge experiments may show how the different cod immune cell subsets respond to immunological challenge, in particular whether the GATA3+ cells could be involved in the B-cell differentiation process. The acquired knowledge will be highly beneficial for the development of antibodies towards cod-specific cell markers, our understanding of alternative vertebrate immune systems and potentially aid cod aquaculture and stock management.

## Data Availability Statement

The sequencing data is available at the ENA repository with project number PRJEB39706.

## Ethics Statement

The rearing and sampling are performed according to animal welfare regulations and approved by the Norwegian authorities (FOTS ID 12336).

## Author Contributions

NG—Data collection and analysis and writing of paper. MS and MB—Data analysis and paper review. SJ and KJ—Intellectual contribution to direction of study and paper review. S-WQ—Intellectual contribution to direction of study, data collection and writing of the paper. All authors contributed to the article and approved the submitted version.

## Funding

This study was funded by the University of Oslo.

## Conflict of Interest

The authors declare that the research was conducted in the absence of any commercial or financial relationships that could be construed as a potential conflict of interest.
